# 

**Published:** 1861-09

**Authors:** 


					﻿;	Publift4ied Hontlily* at per Aiuiuin in Advance.
* ' ■
*5	■'	'	' ' f
X	s
I ’ jOUBNAL. •{
.■
s	■	» .
*■
i
C	•	*
£	.	J. G. WESTMORELAND, M. D., -	‘ A
X ProeesmiI! OF M	Mei-p a a\u Muih ai. JrnispRinESCE in the Athm » Mkiui'al Cdi.i.ei.b,
3	EDITOIi AND PJIDP ItlETo !L	2
?	/Au. ,	■	‘	?
5	Pa X el ^eiei^a. i*erf verita* >.in<* tiinore. •
®	*	'	C
i'^VoL. VHA. «..<EPTE.MB1<Ii, 1«6K	No, I. ? .
! • .
~	A'fLAN'l’A, (4A;	B'
I	A.'mAX'i’^k i?<'rl^r;T,.TOTs.v('KU	• S
-	1 s (5 1 .	•	?
*1
■i	*	•
i	-	- a
«	P2a«>i Xuiuber c-oiitaiiiK Sixty-Four Pai<;eK.
				

